# Child Maltreatment: Skills and Perceptions as Competencies in Higher Nursing Education

**DOI:** 10.3390/children10101607

**Published:** 2023-09-27

**Authors:** Fadwa El Balghity Mouatadir, Jorge Pérez-Pérez, Benito Yañez-Araque, Sagrario Gómez-Cantarino

**Affiliations:** 1 Interventional Radiology Unit, University Hospital (HUT), Health Service of Castilla-La Mancha (Sescam) Toledo, 45071 Toledo, Spain; fadwa-el-balghity@hotmail.com; 2 Faculty of Physiotherapy and Nursing, Toledo Campus, University of Castilla-La Mancha, Avda Carlos III, s/n, 45071 Toledo, Spain; sagrario.gomez@uclm.es; 3 Applied Intelligent Systems Research Group, Department of Physical Activity and Sports Sciences, University of Castilla-La Mancha, Av. Carlos III, s/n, 45071 Toledo, Spain; benito.yanez@uclm.es; 4 Health Sciences Research Unit: Nursing (UICISA: E), Coimbra Nursing School (ESEnfC), 3004-011 Coimbra, Portugal

**Keywords:** child abuse, child maltreatment, child neglect, validation, violence against children, education, public health

## Abstract

Child maltreatment is any action, neglect or aggression towards a child caused by parents, family members or others. The objective of this study is to find out the beliefs and attitudes regarding abuse among nursing students. Methods: This was an exploratory study with the aim of validating the questionnaire, made up of four dimensions. This questionnaire was administered during the 2020/2021 academic year to 370 undergraduate nursing students (first and third year), in Toledo, Talavera de la Reina and Albacete of the University of Castilla-La Mancha, in addition to resident nurses, master’s students and doctoral students during the first four-month period (September, October, November and December). A statistical analysis was carried out observing internal consistency for Cronbach’s alpha. Results: In total, 61.6% of the students concluded that the child was not responsible for maltreatment, and 41.6% thought that it was independent of gender. Furthermore, 65.7% stated that it is human nature for parents to care for their children, 74.1% considered maltreatment to be a crime in the family, and 15.4% said that it does not exist in higher social classes. A total of 23.2% said that those in nursing professions should not intervene in child abuse. With regard to Münchausen syndrome, there was less agreement among the participants, being unknown to the great majority of the students. Conclusions: Among the participants, a lack of knowledge about child abuse was observed, as well as the role of nursing in its detection. Subsequently, given the importance of nursing and its professionals in the detection and management of child abuse, it is necessary to implement knowledge and skills in undergraduate training.

## 1. Introduction

Human behavior encompasses an individual or collective expression of biopsychosocial potential in response to intrinsic and extrinsic stimuli throughout one’s life [1]. While some personality traits remain stable, certain behaviors evolve with life stages, including childhood, adolescence, adulthood, and retirement [1,2].

Child abuse, defined as actions, neglect, or aggression intended to harm or punish a child, with the failure to provide basic needs, is a critical concern [3]. It can be caused by parents, relatives, or others, posing complex physical, psychological, and social risks, affecting children across social strata and ethnicities worldwide [4,5,6].

Child abuse takes various forms, categorized as follows:

1. Physical abuse: Deliberate actions by parents to harm or endanger a child [7].
2. Emotional maltreatment: Actions leading to psychological–psychiatric symptoms that disrupt a child’s development and needs [8].
3. Emotional neglect: The prolonged absence of emotional signals, expressions, and behaviors requiring parental proximity, interaction, and contact [9].
4. Sexual abuse: Involving sexual stimulation or gratification using a child by an adult or another child [10].

Sexual abuse affects one in five women and one in thirteen men, according to the World Health Organization [11]. Neglect occurs when a child’s basic needs are unmet, while other forms of abuse encompass labor exploitation, institutional maltreatment, and Münchausen syndrome by proxy (MSBP) [12]. MSBP, classified as Unspecified Factitious Disorder in the DSM-IV, involves caregivers falsely reporting or fabricating symptoms, and even causing physical harm, typically to assume a sick role for the patient [13]. Suspecting MSBP is crucial for timely intervention in cases involving children. Historically, poverty has led children as young as 5 into labor and girls as young as 6 into household chores, resembling a form of child “slavery” during the 18th–19th centuries [12,13,14,15,16].

All forms of child maltreatment have far-reaching consequences, impacting nervous and immune system development, and increasing the risks of depression, substance abuse, obesity, maladaptive sexual behavior, and unwanted pregnancies [12,17,18,19].

In 1874, a groundbreaking judicial process in the United States initiated global research and legislation efforts, progressively recognizing children’s rights to dignity, privacy, and freedom [20]. The term “battered child syndrome” emerged in the 1960s, accompanied by laws mandating medical professionals to report such cases. In 1989, 146 countries, including Spain, subscribed to the International Convention on the Rights of the Child, ensuring comprehensive protection for children within the family on social, economic, and legal fronts [8,9,21]. In 2006, the United Nations Committee on the Rights of the Child banned all physical punishment and abuse of children. A 2014 UNICEF report indicated that between 500 million and 1.5 billion children endure violence, highlighting the role of child protection laws and the sanctioning of child abuse in raising societal awareness of the issue [22].

Approximately 75% of children aged 2 to 4 (around 300 million) routinely experience corporal punishment or psychological violence from parents or caregivers. Additionally, one in five women and one in thirteen men report childhood and adolescent sexual abuse, occurring from birth to age 17. Furthermore, an estimated 120 million young girls and women under 20 have encountered forced sexual engagement at some point in their lives [23].

In Spain, 5516 cases of domestic violence against individuals under 18 were registered in 2016, with Andalusia having the highest number, followed by Madrid and the Valencian Community. Notably, the Basque Country and Catalonia reported significantly lower numbers of cases, but from 2008 to 2016, child abuse cases in Spain increased, making it a growing social issue [24]. Approximately 5 to 15 out of every 1000 children in Spain experience maltreatment, with sexual abuse affecting 17.9% of children before age 18, including 14.3% before age 13 and 3% at some point in childhood [25]. Healthcare professionals can play a pivotal role in identifying and preventing abuse, but they may lack training and face legal and reporting concerns. A multi-sectoral approach, encompassing caregiver support programs and ongoing family care, is essential for maltreatment prevention. Adequate training and awareness are imperative to address this complex societal problem, considering cultural aspects, social relations, and knowledge transmission [26]. Furthermore, recent statistics reveal a concerning trend. In 2021, there was a significant increase of 37.18% in the number of child abuse notifications compared to 2020, rising from 15,688 to 21,521 cases. Notably, some Autonomous Communities in Spain, such as Navarra (86.29%), Murcia (80.31%), Andalusia (51.16%), and the Balearic Islands (46.88%), experienced substantial increases, while Cantabria saw a notable decrease of 93.30% in notifications compared to 2020. This upward trend warrants attention from nursing professionals [27].

This study aims to contribute to the analysis of nursing students’ education regarding child abuse. It seeks to explore the attitudes and perceptions of future health professionals regarding this issue and assess whether recent changes in nursing education have effectively prepared students for intervention. The primary objective is to validate a questionnaire that analyzes the attitudes, knowledge, and beliefs of nursing students concerning child maltreatment. The study also intends to evaluate various aspects related to child abuse, including the implications for future healthcare professionals in detecting abuse and understanding family dynamics, while also addressing child abuse perpetrated through Münchausen syndrome by proxy.

## 2. Materials and Methods

### 2.1. Objectives of the Study

Based on the incidence of child maltreatment in Spain, the study reveals that 8565 children are currently experiencing various types of abuse, resulting in an annual average of 0.44 cases per thousand. Significant variations exist among different regions, with neglect (79%), emotional abuse (42.5%), and physical abuse (30%) emerging as the most prevalent forms of abuse. Boys are more commonly victimized than girls, comprising 52% of reported cases compared to 47.7% [28]. Nursing assumes a fundamental role in detecting potential cases of maltreatment within the family environment. However, the nursing curriculum lacks substantial training on child abuse. The dearth of research on the knowledge, attitudes, and beliefs of nursing students, along with postgraduate professionals, motivates a statistical study aimed at exploring competencies in child maltreatment [29].

Due to the unavailability of a suitable instrument for conducting the exploratory study, a questionnaire originally designed for pedagogy students has been adapted and tailored for utilization among higher education nursing students [30]. The primary objective is to validate this questionnaire for analyzing nursing students’ attitudes, knowledge, and beliefs regarding child maltreatment.

### 2.2. Study Design and Instrument Validation

The current study involves an exploratory investigation aiming to validate the questionnaire on beliefs and attitudes regarding child maltreatment in higher nursing education. Upon validation, this questionnaire can be employed in both educational and healthcare contexts, adopting a multicenter approach [31]. Survey administration and data collection took place during the academic year 2020–2021, utilizing probability sampling [32]. The questionnaire development involved several stages. Initially, an extensive review of the child abuse literature, with a specific focus on Münchausen syndrome and the nurse’s role in detection, was conducted. Subsequently, a series of items were formulated, delineating the content to be included in the questionnaire: (1) fundamental knowledge of child abuse, (2) education about familial child abuse, (3) nursing implications in child abuse, and (4) comprehension of Münchausen’s syndrome by proxy. To develop these dimensions, we relied on the review of scientific articles where their own research facilitated some of the questions posed. These articles were spread over time and each of these, once reviewed, filled in the gaps that could have occurred in the previous article. Thus, the research carried out in 1996 was of great help, where Chaffin and collaborators [33] studied community data regarding where physical abuse and neglect of children was practiced, after substance consumption by parents and guardians. Added to this research was the study carried out by Moreno Manso [34], which addressed the main theoretical models related to child abuse, carried out in 2006. Important research on child abuse and gender was carried out by Martinez, in 2016 [10], and was used to prepare some items in these dimensions. Regarding nursing implications in child abuse, there was a review of articles that determined the items to be definitively addressed, which were obtained through the research of Merrick and collaborators (2014) [35]. This research addressed child abuse and its prevention from a public health perspective. The question of whether or not nursing professionals should intervene in the event of suspected child abuse was asked. In addition, we evaluated the study carried out by Taylor and collaborators (2015) [36] to prepare another of the items used in this dimension, avoiding a knowledge gap in nursing, where the notification of suspected abuse was discussed. To ensure internal consistency, analogous instruments from akin studies were examined, leading to modifications or adaptations of the initial items. In the fourth dimension, two investigations were reviewed, which were completed over time: the one by Morales-Franco et al. (1995) [37], which deals with infantile Münchausen syndrome, its etiology, diagnostic criteria, and treatment, and the difficulty of its detection, since those who suffer from it do not go voluntarily in search of a solution, and the research Cerda et al., from 2006 [38]. The combination of both investigations generated eight questions, which avoid the existence of knowledge gaps since they address both the symptomatology of children who suffer from this type of abuse, such as the behavior of their parents, and the clinical history in these cases.

The questionnaire underwent scrutiny by experts in statistics, research, and child maltreatment from the Nursing, Industrial Engineering, and Education programs at the University of Castilla-La Mancha. Additionally, group reviews were carried out to attain content validity in the instrument. Following this, prior to initiating a pilot study, questionnaire adjustments were implemented to align with the research objective of understanding beliefs and attitudes concerning child maltreatment within the nursing program [39]. The pilot study served to determine the instrument’s reliability and gauge knowledge, beliefs, and attitudes related to child abuse among Nursing Degree students, specifically in the Child Nursing course at the Faculty of Physiotherapy and Nursing in Toledo (UCLM). Conducted in October 2020 without any issues, this pilot test encompassed 47 participants from the nursing program at the Toledo Campus, lasting approximately thirty minutes. Ultimately, the questionnaire comprised 18 questions distributed across four dimensions (Table 1). Post-pilot test, Cronbach’s alpha coefficient of internal consistency was utilized to ascertain the questionnaire’s reliability.

After statistical analysis, it was concluded that the items with the highest reliability are B002 (most abusers hold the child responsible), B003 (emotional abuse is not as serious as physical abuse), B004 (the consequences of child abuse are different according to gender), and B005 (sexual abuse only affects girls with maladaptive behavior) (Table 2).

On the other hand, questions C004 (child abuse in the family can occur by commission or omission), C006 (parents have mental, alcohol or drug problems with their children), C008 (child abuse in the family is mainly physical), C009 (child abuse in the family decreases as the age of the child increases) and C010 (there is currently a greater social awareness of child abuse in the family) were eliminated. All reformulations were made in order to obtain more reliable results. Therefore, dimension 2 was concentrated into four items (Table 3).

On the other hand, within dimension 3, questions D001 (the role of nursing is fundamental in the detection and notification of child abuse) and D004 (health services are a privileged place for detecting situations of child abuse in the family) were excluded, and D005 (in the health services there should be a protocol for reporting and acting on abuse in the family) was also discarded, being reduced to only two items (Table 4).

The scale related to knowledge of Münchausen syndrome by proxy shows lower internal consistency, a result we associate with the fact that some items do not discriminate, decreasing variability (Table 5 and Table 6).

### 2.3. Participants

Upon reformatting the survey to encompass 18 items, a second round of administration was initiated, engaging first- and third-year nursing students from the Nursing Faculties across the Toledo, Albacete, and Talavera de la Reina campuses of the University of Castilla-La Mancha (UCLM). The questionnaire was also completed by specialized training nurses, inclusive of those in their initial year of residency and the subsequent second year (EIR), master’s degree candidates, and doctoral candidates. The selection criteria encompassed the following: (1) higher education enrollment; and (2) Spanish as the primary language. Exclusion criteria entailed the following: (1) ERASMUS (European Region Action Scheme for the Mobility of University Students) participants; (2) part-time enrolled nursing undergraduates; and (3) distance learning students [40].

A total of 370 higher education students, including both undergraduates and postgraduates from UCLM, along with specialist nurses (EIR) within the health service of Castilla-La Mancha (Sescam), partook in the study, yielding an 86.85% response rate. All participants fulfilled the inclusion criteria, with the majority aged between 18 and 30 years (mean = 21.25; SD = 4.77), with variations in the student cohort (ranging from a minimum age of 18 among undergraduates to a maximum of 68 among doctoral candidates). The sample size was optimal for questionnaire validation, meeting the minimum requirement of 300 students as expressed by Comrey in his study carried out in 2013 [41]. As for participant gender distribution, 68 individuals (18.4%) identified as male, while 302 (81.6%) identified as female, completing the questionnaire.

Regarding participants’ academic level, the study encompassed 349 undergraduate students, with 91 in their first year (24.59%) and 258 in their third year (69.7%). Additionally, 21 postgraduate students participated, comprising 8 master’s degree students (2.2%), 6 first- and second-year specialized nursing residents (8%), and 7 doctoral candidates (1.9% of the total). It is important to emphasize that this choice was made because in the first year of nursing, the knowledge that the students have before starting university studies is assessed. By exploring the same question with the third-year students, we try to reflect the acquisition of knowledge and a more specific assessment of the subject. In this way it is assessed whether the curricula and teachers are sufficient for the acquisition of knowledge. On the other hand, the choice of postgraduate students is identified by the need to find out whether they know the subject, whether they are trained in it and how they would approach it when immersed in clinical practice. In this way, a more professional and less “apprentice-like” vision is promoted.

As for the participants, it is important to note that, demographically, most of them carry out their care practice within the same Health Area (No.1), so the care units, both urban and rural, have a unification of criteria in the field of care management. In addition, this research will allow us to deepen our understanding of the knowledge that students of higher nursing degrees have about child abuse in the UCLM (Toledo), governed by the same cultural pattern in Castilian-La Mancha, Spain.

### 2.4. Procedures

The questionnaire was administered at the UCLM facilities, in paper format, by a teacher, coinciding with a master class (undergraduate students), a clinical session (specialist nurses) and a work meeting (doctoral students). It was individually completed under the supervision of the teacher and in a period of approximately thirty minutes, after informing the students about the object of the research, in addition to the signing of the informed consent form, where the students were warned of the non-existence of economic and academic incentives. The entire procedure to be followed was explained by the researchers, and compliance was achieved through continuous supervision. To ensure equal opportunities, the data collection instrument was given to both women and men, without any distinction on the basis of gender, thus guaranteeing fairness.

### 2.5. Ethics Approval

This study was approved by the Ethics Committee of the Complejo Hospitalario de Toledo (CHT) Spain, under number 01720-83CHT. All study participants were informed verbally by the principal investigator of the reason for the research and this information was included in the informed consent form, which was signed before participating in the study. Participants were also informed that they could stop completing the questionnaire at any time. In addition, members of the research team signed a confidentiality document. This research respects the fundamental principles of the Declaration of Helsinki, the Declaration on Human Rights and Biomedicine of the Council of Europe, the Universal Declaration on the Genome and Human Rights of UNESCO and the Oviedo Council on Human Rights and Biomedicine. All participants’ data have been treated confidentially in accordance with Organic Law 7/2021, 26 May, on the protection of personal data processed for the prevention, detection, investigation and repression of criminal offences and the execution of criminal sanctions, keeping them strictly confidential and not accessible to unauthorized third parties.

## 3. Results

We will proceed to present the results obtained after completing the questionnaire on beliefs and attitudes towards child maltreatment, and carry out a statistical study of the data using the SPSS version 24 computer system.

With regard to “knowledge of basic concepts about child abuse” in the first dimension of the questionnaire and according to the results obtained, 61.6% of pupils think that the child is not responsible for the abuse. However, 19.2% (71 students) think the opposite. We even find that the same number of pupils do not define themselves in this sense.

It was also considered important to investigate the gender-related consequences of child maltreatment. In this sense (Table 7), 41.6%, corresponding to 154 students, believe that maltreatment does not affect one sex or the other to a greater extent. However, 31.1% think that child maltreatment is determined by the sex of the children. It is worth noting that 27.3% (101) took an indeterminate position. Moreover, it is striking that, when relating sexual abuse to inappropriate behavior in girls, 33 students answered in the affirmative. It is also striking that 39 students did not define themselves in this respect, a percentage that together amounts to 19.4%. This confirms the magnitude of the problem and the need for training on the subject.

Taking into account the results of the second dimension (Table 8) regarding education about child abuse in the family environment, degree of interest and knowledge of the student, it stands out that in the question where it is indicated that human nature drives parents to care for and love their children (C001) 65.7% agree with this affirmation. It is worth noting that 34.3% say the opposite and do not even take a position. However, 74.1% consider that child abuse is a crime in the family. In fact, 15.4% of respondents said that child abuse does not exist in the upper social classes. This situation highlights the need for specific training related to child abuse in the family nucleus.

Nursing plays a vital role in detecting and preventing child abuse in both primary care and specialized care settings. In total, 23.2% of students believe that those in nursing professions should not intervene when faced with a suspicion of maltreatment (D002), while 1.4% are undecided. However, 75.4% of students agree on the need to intervene and 77% are willing to report any possible negligence towards children to the health service or to the user (D003).

The last aspect of the questionnaire is related to the pupils’ knowledge of Münchhausen syndrome by proxy (Table 9). Within this dimension, we ask the question of whether the child expresses atypical symptoms in a persistent manner, leading to a disordered diagnosis (E002). It can be seen that 60% agree with the question posed, while 9.8% disagree, and 30.3% give a neutral response. In this syndrome by proxy, the perpetrator intentionally produces or feigns physical and even psychological symptoms and signs in another person in their care (usually an infant). Therefore, finding that 40.1% of pupils think that this situation is not correct, and even stay away from it, leads us to believe that there is a significant lack of knowledge on the subject.

One of the questions with the greatest degree of dispersion in the results is that referring to “the mother or father avoids leaving the child unaccompanied in hospital (E004)”, with which 31% of students agree. However, 39.2% disagreed and even 30% of the students were neutral. Münchausen syndrome is usually provoked in most cases by the mother figure of the abused child.

We can see that the results are more conclusive for the question related to the signs and symptoms suffered by the child only occurring in the presence of the mother or father (E005). We found that more than 60% of the sample agreed on this question. However, 7.3% disagreed.

It is also interesting to highlight the results obtained in relation to the knowledge of health and specific care that parents provide for the child, despite not being health professionals (E006). It was found that 38.9% disagree with this question, with only 29.2% who think that this situation is true. On the other hand, it should be noted that 40% thought that the child could be subjected to in-hospital techniques without need (venipuncture, administration of drugs, etc.), while only 18.9% disagreed with this. It was found that 41.1% are neutral on this serious issue.

It can be affirmed that in this fourth dimension there is a fluctuation of students in neutral and disagreement responses, a situation that may indicate the need for formal training within the training plans of higher education in nursing about Münchausen syndrome by proxy.

## 4. Discussion

In this study, the researchers employed a survey to be validated within the context of higher education, specifically targeting health professionals, particularly nurses. The survey aimed to explore various dimensions related to child abuse, including attitudes, beliefs, necessary training, and practical approaches within the field. It sought to establish a constructive dialogue with both undergraduate and postgraduate students, equipping them with the essential skills to address child abuse effectively.

Child maltreatment has been a topic of formal conceptualization for the past five decades. Despite this, limited awareness and understanding persist, particularly among nursing professionals. The study investigated 370 nursing students from diverse educational levels, including undergraduates, postgraduates, and specialized training programs. The majority of participants were third-year undergraduates, around 20 years old. Findings from the study revealed that while students possessed fundamental knowledge of child abuse, significant disparities existed in their understanding of different maltreatment types and their consequences. This highlighted a concerning gap in the preparation of educational professionals, echoing concerns raised by Goldman and Grimbeek in 2011 [42].

One of the key findings of this study was the perspective on the accountability of child victims. Approximately 61.6% of nursing students believed that the child, as a victim, should not be held accountable for experiencing maltreatment, while 19.2% held contrary views. Additionally, this study explored gender dynamics concerning child abuse, with 41.6% of students believing that child abuse did not disproportionately affect a particular gender, while 31.1% disagreed. These disparities in perspectives partially stemmed from explanatory models of child maltreatment dating back to the 1970s, which examined the victim–perpetrator relationship and often attributed it to the personality traits of the maltreater. This emphasized that the child was not responsible for maltreatment, but coexisting with an individual exhibiting destabilized behavior increased the risk of such acts [13,43].

This study also addressed the misconception that child maltreatment might be perceived as corrective or instructive due to altered parental expectations. It stressed the importance of avoiding mistreatment in education. Historical practices, such as those observed in Jericho, where children with impairments were confined to structures, were cited as examples of misguided notions [18].

Furthermore, the study delved into gender differentials in child maltreatment. While these differentials were complex, distinct gender disparities were observed in child sexual crimes, primarily affecting women due to cultural associations with sexuality. This perpetuated the objectification of women, and the study highlighted instances of mistreatment faced by victims of sexual assault by their relatives [38]. A sociological framework from the 1970s emphasized the role of cultural, social, and familial factors as crucial triggers of child abuse [44].

The research also explored nursing students’ knowledge regarding child abuse in the family environment. It revealed that intra-family child abuse was a recurring practice with a high incidence of psychosocial consequences for minors [45]. The Childhood Observatory defined child abuse within the family as any action exercised in a pediatric case by a relative of the minor, including parents, siblings, grandparents, etc. [46]. The study indicated that 74.1% of nursing students considered child abuse in the family as a crime, while 21.6% had opposing views. Interestingly, 87 students (23.5%) believed that basic needs should not be covered by parents, and 10.8% remained undecided on this matter. It was noted that the subject of child nursing was introduced in the third year of nursing degree syllabuses, possibly explaining the lack of consensus among first-year students. Despite the complexities, it is evident that formal training in specific competencies related to child rearing and continuous care of the family environment could help detect and prevent child abuse [13].

The study also highlighted the high degree of mistreatment within families, emphasizing the responsibility of future professionals within the health services [47]. Hospitals were often the primary locations for treating infants with injuries resulting from child abuse, underscoring the significance of detecting and reporting such cases [13,48]. The study drew upon theories from the school of behavioral psychology, which analyzed human behavior, to explain the relationship between parents’ emotional hyper-reactivity to their children’s misbehavior and child abuse [13,49].

The research findings underlined the importance of assessing nursing students’ awareness of their role in addressing child maltreatment [50,51,52]. A study conducted in the Spanish context by Soy-Andrade et al. [53,54,55,56] demonstrated the benefits of direct educational strategies in this regard. It was essential for nursing students to recognize the need for involvement and formal notification in cases of suspected child abuse, highlighting the critical role of nursing staff in intervening appropriately. Spain’s protocol for addressing child abuse called for effective intersectoral work, with two lines of notification—one ordinary and another urgent—depending on the level of imminent danger faced by the child [54,57,58].

This study also examined the relatively unknown syndrome of Münchausen by proxy or factitious disorder inflicted on another, as defined by the Diagnostic and Statistical Manual of Mental Disorders (DSM-5) [59]. This syndrome involves a subject simulating symptoms suffered by another person, typically a child, resulting in serious health consequences. The study revealed that a significant percentage of students (30.3%) were unaware that the symptoms suffered by the child were caused by their parents. Münchausen by proxy is characterized by a pattern of self-aggression or harm toward others, where the perpetrator fabricates symptoms, signs, and illnesses in the victim, often a child [60,61]. This leads to severe health issues in the child, highlighting the serious nature of this form of child abuse [62,63]. Furthermore, the study found that 30.5% of students were unaware that it was typically women, often mothers, behind these cases. While it is true that nearly 95% of such cases involve mothers as the abusers, it was emphasized that these mothers tend to have some knowledge of health-related matters, often studying health sciences, albeit without completing their studies [64].

This study underscored that this situation facilitated both the execution and continuation of abuse when children were admitted to hospitals, as these mothers tended to establish trust with the healthcare staff and rarely left their children alone in hospital units [65]. Incidence rates for this syndrome have been cited, showing that it is a rare but serious problem. In the United Kingdom, it has an incidence of approximately 2.8 cases per 100,000 in children under 1 year of age and 0.5 cases in children over 16 years of age. In the USA, it has reached about 200 new cases annually, affecting both sexes equally [66]. This syndrome is associated with a high mortality rate, and most deaths occur within hospital settings despite efforts by healthcare teams to make accurate diagnoses [67].

Given the infrequent nature of this syndrome, it often goes unnoticed or unknown, making it critical for healthcare professionals, particularly nurses, to be aware of its existence and manifestations [68]. Nursing curricula in Spain typically comprise 240 credits under the European Credit Transfer System (ECTS), with some autonomy for universities to define course content. The World Health Organization’s recognition of the need to train nurses in the field of child abuse has led to the gradual inclusion of relevant content in study plans [69,70].

This study highlighted the importance of competence in identifying and managing child abuse and neglect (CAP) for health professionals. It noted a deficiency in understanding, likely attributable to insufficient training. Furthermore, the level of knowledge and attitudes of health professionals toward training in this field have not been extensively researched [70,71]. To address these gaps, the nursing curriculum at the University of Castilla-La Mancha (UCLM-Enfermería, Toledo) incorporated child abuse training, particularly in the subject of child nursing during the third year. This training consisted of 9 h, delivered by healthcare professionals with expertise in the field. Similar initiatives are being adopted in other university campuses in the region (UCLM) and other autonomous communities, including in postgraduate education.

This study indicated that nursing students themselves expressed dissatisfaction with the teaching provided through the curricula of medicine and nursing careers, citing it as insufficient. For instance, a study in Greece revealed that only 19.7% of participants reported that their study plan included a chapter dedicated to NAC, while 27.3% mentioned that a teacher had initiated discussions on the topic. Similar findings were documented in research conducted in China, the United States, and Saudi Arabia. Notably, students in the clinical stages of their medicine and nursing programs lacked confidence in managing suspected CAP cases, possibly due to their perception of inadequate knowledge. In contrast, those in the preclinical stages might not fully grasp the complexities of NAC, potentially underestimating its intricacies. Despite these challenges, a significant proportion (80.8%) of respondents felt moderately prepared or less to deal with suspected CAP cases in their future professional practice. These trust deficits mirrored similar findings in research involving health professionals in Greece and other international settings, emphasizing the need for a systematic and structured approach to training future healthcare professionals in this field. A proposed model aimed at enhancing the understanding of physicians and other health professionals about child physical abuse through the training of trainers and workshop modules was highlighted as a potential solution [69,70,71].

It is essential to acknowledge certain limitations of this study, including potential refusals by some students to complete the questionnaire. To address this, a 10% replacement rate for the sample size was established. Additionally, we recognize limitations related to the instrument itself, which may have restricted the collection of other relevant variables. The individual completion of questionnaires in the presence of a researcher/teacher may have caused discomfort for some students. It is important to note that the study was conducted in one autonomous community, Castilla-La Mancha, Spain, indicating the need for further research to bolster the study’s findings and inform higher education teaching systems more broadly [13,42,47,69,70,71].

## 5. Conclusions

The European Credit Transfer System (ECTS) formulated the curricula of Higher Education in Health Sciences to include a total of 240 credits. This situation has generated a new paradigm in the method of learning, where students acquire important knowledge in their own training. This situation has led to advances in healthcare.

However, there is still no generalized unification of criteria within training in the health sciences for the teaching of some specific concepts that incorporate the issues learned about into healthcare practice. In this sense, with regard to child maltreatment, more training is necessary during undergraduate studies, continuing this focus into postgraduate studies.

Prioritizing training and practical experience in higher education in nursing studies equips students with the necessary competences to deal with real situations in healthcare practice. Thus, by fostering knowledge about child abuse in the classroom through theoretical training and seminars where learned skills are strengthened, new knowledge is assimilated, the fear of making mistakes is avoided, and the lack of practical experience is remedied. This type of education generates more personalized care for children and their families by future professionals, as well as healthcare staff immersed in care practice. In fact, the use of tools such as questionnaires is necessary in higher education, as they are used to identify attitudes, beliefs, and gaps in training. In this way, new forms of learning can be managed from the undergraduate level so that teachers involved in different training subjects can promote knowledge and skills that facilitate practical care activity, in order to respond appropriately to the detection of possible cases of child maltreatment.

For future health professionals, as well as those practicing in healthcare settings, it is important that they have the unique ability to detect cases of child maltreatment, a global problem that directly affects the health and well-being of children.

The managers of health policies should include improvements that facilitate continuous training on this issue, and the inclusion of different units and services of health personnel specifically trained in dealing with this issue.

Therefore, future health professionals should acquire specific knowledge within their training period through seminars, simulated clinical cases, and social health lectures, so that they, like other professionals in the healthcare team, can actively participate in care with a greater level of involvement. Thus, child-centered care will become safer, more efficient, effective, and timely. Future research could investigate the experiences of student nurses in relation to child maltreatment.

## Figures and Tables

**Table 1:** Dimensions that make up the questionnaire.

Dimensions No. of Questions
Basic knowledge of child maltreatment, Questions 1–4
Training on child maltreatment in childhood, Questions 5–8
Nursing in child maltreatment, Questions 9–10
Knowledge of Münchausen’s syndrome by proxy, Questions 11–18

**Table 2:** Results of Cronbach’s alpha internal consistency coefficient for the dimension of basic knowledge about child maltreatment.

Dimension 1	Correlation Corrected Total Element	Cronbach’s Alpha if the Element Is Removed
Most abusers hold the child responsible for this (B002).	0.584	0.730
Emotional abuse is not as serious as physical abuse (B003).	0.720	0.650
The consequences of child maltreatment differ according to gender (B004).	0.507	0.775
Sexual abuse only affects girls with maladaptive behavior (B005).	0.560	0.742

**Table 3:** Results of Cronbach’s alpha internal consistency coefficient for the dimension of training on child maltreatment in the family.

Dimension 2	Correlation Corrected Total Element	Cronbach’s Alpha if the Element Is Removed
Human nature drives parents to care for and love their children (C001).	0.726	0.860
Child abuse in the family is a private matter (C002).	0.699	0.870
Child abuse in the family is a crime (C003).	0.815	0.826
Child abuse in the family does not exist in the upper social classes (C005).	0.763	0.847

**Table 4:** Results of Cronbach’s alpha internal consistency coefficient for the nursing dimension in child maltreatment.

Dimension 3	Correlation Corrected Total Element	Cronbach’s Alpha if the Element Is Removed
Nursing should not intervene when abuse is suspected (D002).	0.820	0.860
Any person (professional or not) should report a suspicion of abuse (D003).	0.820	0.860

**Table 5:** Results of Cronbach’s alpha internal consistency coefficient for the dimension of knowledge of Münchausen syndrome by proxy.

Dimension 4	Correlation Corrected Total Element	Cronbach’s Alpha If the Element Is Removed
At the household level, there is the possibility of parents’ omission of care for the child, compromising the child’s health (E001).	0.368	0.628
The child expresses symptoms in a persistent and atypical manner, leading to a disordered diagnosis (E002).	0.494	0.597
During the clinical interview, disorders in the parent–child relationship were observed (E003).	0.349	0.633
The mother or father avoids leaving the child unaccompanied in hospital (E004).	0.309	0.641
The child’s signs and symptoms only occur in the presence of the mother or father (E005).	0.268	0.556
Parents have knowledge of health and child-specific care, even though they are not health professionals (E006).	0.298	0.647
In this case, the child may be subjected to unnecessary in-hospital techniques (venipuncture, drug administration, etc.) (E007).	0.385	0.622
Diagnostic tests performed are not consistent with the child’s health status (E008).	0.401	0.621

**Table 6:** Results of Cronbach’s alpha internal consistency coefficient for the dimensions of the questionnaire.

Dimensions	Cronbach’s Alpha	No. of Items
Basic beliefs about child maltreatment	0.784	4
Attitudes to child maltreatment in the family	0.885	4
Nursing attitude in child abuse	0.901	2
Knowledge of Münchausen syndrome by proxy	0.671	8

**Table 7:** Dimension 1: Basic knowledge of child maltreatment.

The Child Is Responsible for the Maltreatment (B002)			Child Maltreatment in Relation to Sex (B004)			Sexual Abuse: Inappropriate Behaviors in Girls (B005)		
Disagreement	Neutral	Agreement	Disagreement	Neutral	Agreement	Disagreement	Neutral	Agreement
19.2%		61.6%	41.6%	27.3%	31.1%	80.6%	10.5%	8.9%

**Table 8:** Dimension 2: Knowledge about child maltreatment in the family environment, degree of interest and knowledge of the student.

Parental Care for Children Is Innate (C001)			Child Abuse in the Family Is a Crime (C003)			Child Maltreatment Does Not Occur in the Upper Social Classes (C005)		
Disagreement	Neutral	Agreement	Disagreement	Neutral	Agreement	Disagreement	Neutral	Agreement
23.5% (87)	10.8% (40)	65.7% (243)	21.6% (80)	4.3% (16)	74.1% (274)	77.1% (285)	7.6% (28)	15.4% (57)

**Table 9:** Dimension 4: Knowledge of pupils regarding Münchausen syndrome and Münchausen syndrome by proxy.

Dimension	Strong Disagreement	Disagreement	Neutral	Agreement	Fully in Agreement
The child expresses symptoms in a persistent and atypical manner, leading to a disordered diagnosis (E002).	4.1%	5.7%	30.3%	47.8%	12.2%
The mother or father avoids leaving the child unaccompanied in hospital (E004).	1.9%	5.4%	23.2%	49.7%	19.7%
The child’s signs and symptoms only occur in the presence of the mother or father (E005).	11.1%	28.1%	29.7%	23.2%	7.8%
Parents have knowledge of health and child-specific care, even though they are not health professionals (E006).	12.4%	26.5%	31.9%	23%	6.2%
In this case, the child may be compromised by unnecessary in-hospital techniques (venipuncture, drug administration, etc.) (E007).	6.5%	12.4%	41.1%	29.2%	10.8%
Diagnostic tests performed are not consistent with the child’s health status (E008).	2.2%	8.9%	45.4%	34.9%	8.6%

## Data Availability

The data used in this study can be obtained upon reasonable request to the corresponding author.
